# Thermal Characterization of New 3D-Printed Bendable, Coplanar Capacitive Sensors

**DOI:** 10.3390/s21196324

**Published:** 2021-09-22

**Authors:** Mattia Alessandro Ragolia, Anna M. L. Lanzolla, Gianluca Percoco, Gianni Stano, Attilio Di Nisio

**Affiliations:** 1 Department of Electrical and Information Engineering, Polytechnic University of Bari, 70126 Bari, Italy; anna.lanzolla@poliba.it (A.M.L.L.); attilio.dinisio@poliba.it (A.D.N.); 2 Department of Mechanics, Mathematics and Management, Polytechnic University of Bari, 70126 Bari, Italy; gianluca.percoco@poliba.it (G.P.); gianni.stano@poliba.it (G.S.)

**Keywords:** additive manufacturing, fused filament fabrication, conductive filaments, capacitive level sensors, flexible sensors, thermal characterization

## Abstract

In this paper a new low-cost stretchable coplanar capacitive sensor for liquid level sensing is presented. It has been 3D-printed by employing commercial thermoplastic polyurethane (TPU) and conductive materials and using a fused filament fabrication (FFF) process for monolithic fabrication. The sensor presents high linearity and good repeatability when measuring sunflower oil level. Experiments were performed to analyse the behaviour of the developed sensor when applying bending stimuli, in order to verify its flexibility, and a thermal characterization was performed in the temperature range from 10 °C to 40 °C to evaluate its effect on sunflower oil level measurement. The experimental results showed negligible sensitivity of the sensor to bending stimuli, whereas the thermal characterization produced a model describing the relationship between capacitance, temperature, and oil level, allowing temperature compensation in oil level measurement. The different temperature cycles allowed to quantify the main sources of uncertainty, and their effect on level measurement was evaluated.

## 1. Introduction

Additive manufacturing (AM) technologies appear to be very appealing for the fabrication of sensors: in particular, the two main classes of AM-based sensors can be summarized as follows: sensors for engineering applications and sensors for medical applications [1]. Among all the different AM technologies, material extrusion-based technologies, are very appealing for sensor fabrication due to several benefits such as the possibility to print more materials in the same working cycle (to switch printing mechanisms when going from one material to another one is a very challenging topic, and several solutions have been implemented [2]). Fused filament fabrication (FFF) technology is widely employed in this field and different studies were developed to correlate process (and post-processing) parameters with sensor performance, in particular to improve the conductivity of 3D-printed tracks [3,4,5,6]. Generally, FFF is extensively employed to manufacture piezoresistive-based sensors, ranging from accelerometers [7] to force and motion sensors [8,9,10], allowing the fabrication of systems with integrated sensors and actuators [1,2], enabling technology development in forefront fields such as the Internet of Things and the Internet of Robotic Things [11].

As the state of the art, sensors manufactured through FFF technology for temperature sensing are based on the thermoresistive effect: changes in external temperature are detected by means of sensor resistance changes. As a matter of fact, the extruded conductive materials employed for these purposes are plastic-based materials doped with conductive elements (i.e., carbon nanotubes, carbon black, etc.) and the principle underlying the relation between temperature and resistance is complex and not fully understood (negative and positive temperature coefficients have been found for the same material at different temperatures). Several interesting studies [4,12,13,14,15] were carried out to characterize the resistance-temperature dependence: in the current the state of the art, these sensors require deeper studies and do not show a good repeatability, making them unusable for industrial applications. It is thus necessary to study the reliability of 3D-printed devices, as well as to improve their repeatability [16,17]. In fact, reliability evaluation has a crucial importance in the characterization of measurement systems [18,19].

In the proposed study, FFF technology has been used to fabricate bendable coplanar sensors based on the capacitive principle, which have been characterized in terms of no sensitivity to bending stimuli, and from a thermal point of view, by using an industrial climatic chamber. The sensor structure described in this paper has been designed and demonstrated for measuring liquid level in a tank by sensing the change of capacitance. One key advantage of the proposed technique is that the sensor may also be directly integrated in a liquid container fabricated with additive manufacturing. Moreover, it should be apparent that the fabrication of the capacitive sensor on a flexible substrate may be useful for applications where rigid sensors are not suitable, such as soft robotics and wearable sensors. For example, capacitive sensors can be exploited for force and tactile sensing in artificial skin [20]. Other applications may involve the detection of liquid or the measurement of liquid level in flexible bags and tubing for medical use, or the detection of liquid leakage.

## 2. Manufacturing

The FFF extrusion-based AM technology was employed to manufacture the proposed coplanar, capacitive sensors. The Ultimaker 3 (Ultimaker B.V., Utrecht, Netherlands) dual extruded 3D printer used offers the following advantages:

(1) The possibility to directly extrude two different materials in the same printing cycle. Thermoplastic polyurethane (TPU 95A, Ultimaker B.V., Utrecht, Netherlands) and a conductive material (AlfaOhm, Filoalfa by Ciceri de Mondel S.r.l., Turin, Italy) were employed for the flexible substrate and the electrodes, respectively. According to corresponding technical data sheets, the first one is characterized by an elongation at break of 580%, whereas the second one is a polylactic acid (PLA)-based filament doped with carbon nanotubes with a resistivity of 15 Ω cm along the layers.
(2) The usage of only one manufacturing technology to manufacture the whole sensor: generally, at least two different technologies are required (one for the electrodes and one for the flexible substrate), so a reduction of manual and assembly tasks was achieved in conjunction with a reduction in the supply chain.

The fabricated sensor thus consisted of a flexible substrate and two electrodes. Moreover, a flexible cover (made of TPU 95A) was placed over the electrodes to isolate them from the surrounding environment.

**Figure 1:**
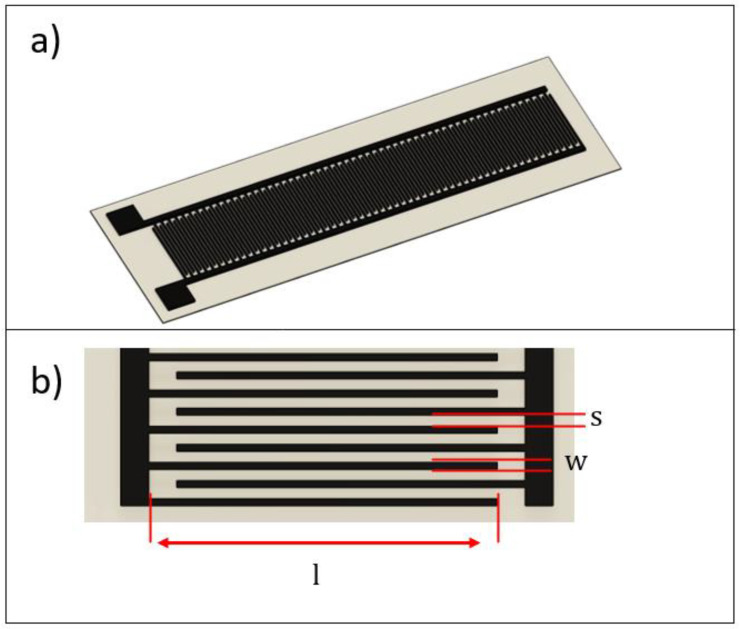
CAD model of the capacitive sensor: (**a**) design of the whole sensor, and (**b**) overview of sensor’s main parameters.

The main design parameters are listed in Table 1: they refer to Figure 1b, and to Equations (1) and (2) described in Section 3.1. A design for additive manufacturing (DfAM) approach was used. The electrode width (w) was set as a function of the employed nozzle size (namely 0.4 mm). Since the electrode was manufactured from a single extruded rod, it was chosen to set the w parameter slightly higher compared to the nozzle size to increase the fluidity of the melt filament coming out of the nozzle. With regard to the second major design parameter, i.e., electrode spacing (s), it has been found by a trial-and-error approach that 0.8 mm is the minimum value to avoid cross contamination among two adjacent electrodes. Other parameters, such as the number of electrode pairs (*N*), electrode height and flexible top coverage height can be arbitrarily chosen as a function of the specific application.

**Figure 2:**
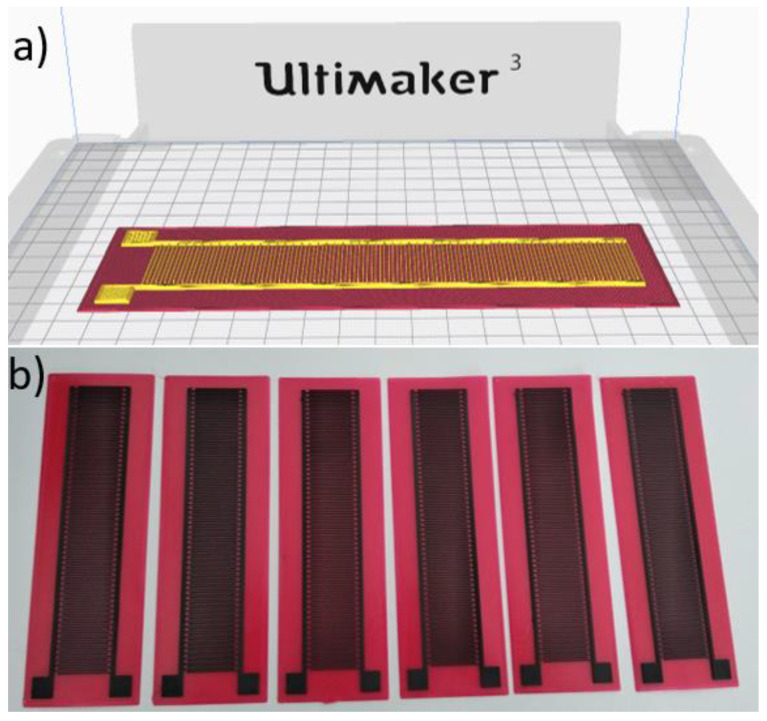
Fabrication of the sensor: (**a**) representation in the slicing software, and (**b**) six manufactured sensors. The top silicon layer has not been printed in this production batch.

The slicing software Ultimaker Cura 4.6 was used to slice the CAD model and generate the g.code file to send to the 3D printer. A dual extruder 3D-printing process is hard to manage due to several problems such as cross contamination, breakdown problems of the conductive materials etc., consequently particular attention was paid to the process parameters. 0.4 mm and 0.8 mm nozzles were used for the AlfaOhm and TPU 95 A materials, respectively; moreover, (i) a layer height of 0.2 mm was set for both materials to reduce the total printing force [21], thus reducing breakdown problems for the conductive filament, and (ii) low printing speeds were employed to reduce cross-contamination problems. A line width of 0.5 mm (parameter *w* in Table 1) was set, bigger than the nozzle diameter of 0.4 mm, to avoid the common underextrusion problems experienced when conductive materials are used. The total cost of the sensor, computed by the slicing software considering the cost per meter of the initial raw material and the length of the extruded filament, was 0.38 €, whereas the printing time, computed by the slicing software as well, was 56 min. The main process parameters are shown in Table 2, whereas the sensor as represented by the slicing software and several manufactured sensors are shown in Figure 2. After the printing, electrical wires were soldered to the sensors’ pads at a temperature of 200 °C.

## 3. Characterization

The proposed bendable coplanar capacitive sensor is a novelty from a manufacturing point of view. The sensor can be used for liquid level measurement, as will be shown here for the case of sunflower oil. In this study, the sensor has been tested: (i) for different applied bending stimuli, to highlight the absence of capacitance variations, and (ii) with thermal cycles to understand the relationship between capacitance and level at different temperatures. All tests have been performed after zeroing the offset capacitance of the multimeter and leads.

### 3.1. Bending

Growing interest has emerged over the last few years in the field of 3D-printed flexible sensors: AM-based technologies seem to fit very well with the sensors’ requirements, leading to several advantages from a manufacturing point of view (i.e., reduction in the number of assembly tasks, huge geometric freedom, etc.). Extrusion-based methods are mainly used to fabricate flexible and wearable sensors based on piezoresistive effects [22], where movements are detected by a change in resistance. Generally, 3D-printed wearable sensors are used to measure quantities related to human body movements such as knee bending, hand movements, etc. [23]. The proposed sensor, instead, has been tested to investigate its potentialities as a wearable sensor unrelated to movement.

Unlike previously described sensors, in this case the sensing mechanism is not based on a change of resistance but on a change of capacitance.

The constitutive equation for coplanar capacitive sensors is as follows [24]:

(1) $$C=Nl\varepsilon_{0}\varepsilon_{ea}\frac{K(\sqrt{1-k_{0}^{2})}}{K\left(k_{0}\right)}$$

where C is the capacitance of the whole sensor, N is the number of electrodes pairs, l is the length of each electrode,$\;\varepsilon_{0}$ is the vacuum permittivity, $\varepsilon_{ea}$ is the effective permittivity of the capacitive sensor in the air (further details about this parameter are well explained in [24]), and $K\left(k_{0}\right)$ is the elliptical integral of the first kind in terms of $k_{0}$, where $k_{0}$ is defined as follows:

(2) $$k_{0}=\frac{s}{s+2w}$$

where s and w are the electrodes spacing and width, respectively. The main design parameters, described in Equations (1) and (2) are shown in Figure 1. The predicted capacitance of the sensor, obtained from Equation (1), is 82.7 pF.

**Figure 3:**
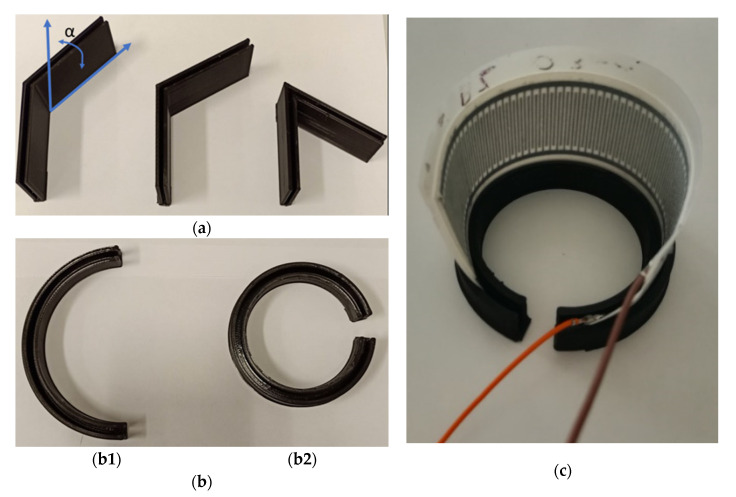
Experimental set-up for analysis of sensitivity to bending: (**a**) bending supports with α of 45°, 60° and 120°, (**b**) two C-shaped bending supports (indicated as **b1** and **b2**), and (**c**) installation of a sensor on the b2 bending support.

The correlation between capacitive changes and applied bending stimuli was studied to investigate the suitability of the proposed sensors in the field of the wearable sensing. The sensor was bended by means of 3D-printed custom-made supports: two different kinds of supports were used, as shown in Figure 3. The first kind of supports provided a bending angle α of 45°, 60° and 120°, whereas the second one was composed of two C-shaped supports (indicated as b1 and b2) which bent the whole sensor with a constant radius of curvature.

The sensor was placed into each bending support for 3 min: no significant change in sensor capacitance was detected, compared to the initial capacitance value without bending supports. The mean of 100 readings of capacitance value is listed for each bending support in Table 3. The standard deviation (STD) of the mean value resulted lower than the resolution of the multimeter. It must also be noted that the capacitance of the 3D-printed sensor differs from the one obtained by applying Equation (1). In fact, it must be considered that Equation (1) results from an approximated model; moreover, an error in the actual parameters always occurs in the FFF process (due to mechanical vibrations, lack of uniformity in raw materials, environmental conditions and so on), causing a mismatch between the designed sensor and the printed one.

The insensitivity of the proposed sensors to bending stimuli is an interesting aspect which lays the foundation for their exploitation in the field of wearable sensing: they could be used in applications where a quantity is measured despite bending perturbations are applied.

### 3.2. Thermal Characterization

**Figure 4:**
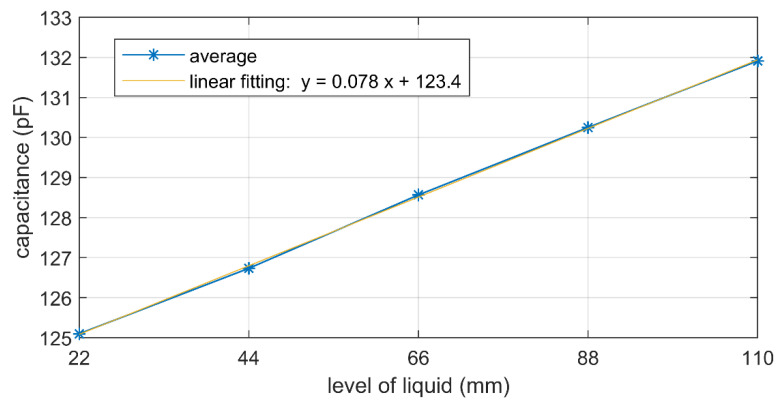
Capacitance vs. level of sunflower oil. The reported capacitance is the average of 10 tests, where in each test the tank is filled with 5 oil levels from 22 mm to 110 mm.

The developed sensor was inserted into a 3D-printed tank, with dimensions of 73 mm, 43 mm and 166 mm along *x*-, *y*-, and *z*-axis, to be filled with sunflower oil. Preliminary experiments were conducted to assess the dependence of capacitance on liquid level at room temperature, by filling the tank with 22, 44, 66, 88 and 110 mm of sunflower oil. A good linearity was observed, as shown in Figure 4, obtaining a sensitivity of 0.078 pF/mm. Maximum deviation from linearity was 0.9% of the range represented in that figure.

The linear characteristics of these capacitive sensors for liquid level sensing could be affected by the temperature of the liquid and the environment, hence it becomes necessary to analyse the relationship between temperature and capacitance to compensate measurement errors in liquid level sensing. In this section the temperature dependence of the capacitance of the manufactured coplanar capacitive sensors has been analysed in a wide temperature range.

**Figure 5:**
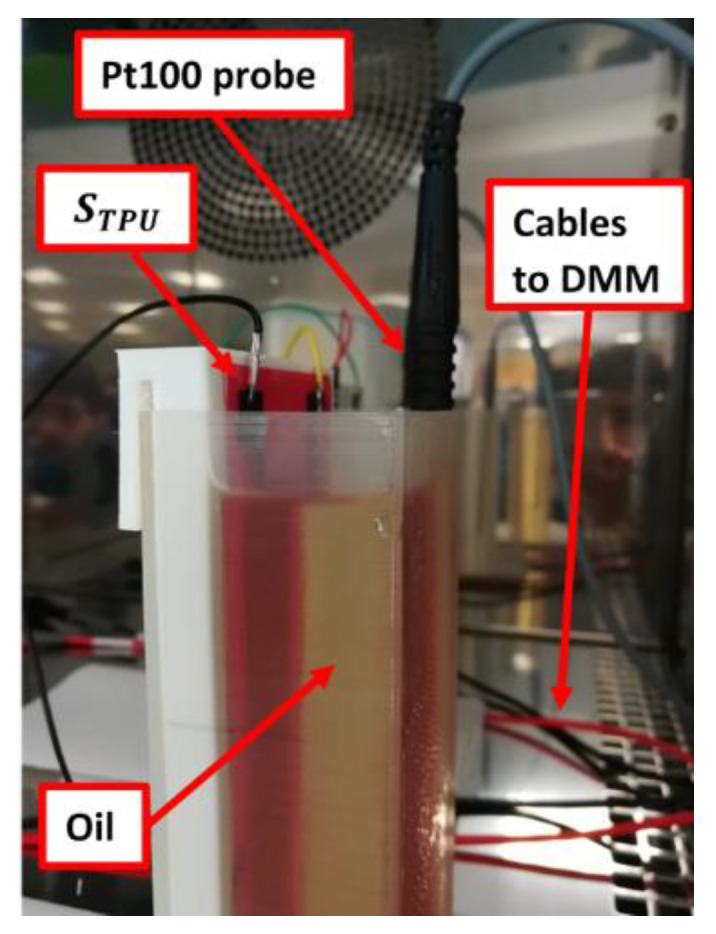
Experimental set-up for thermal characterization inside a climatic chamber. The sensor, $S_{\mathrm{TPU}},\;\;$ is installed inside an oil tank. Oil temperature is measured with a Pt100 probe.

The experimental setup, shown in Figure 5, is composed of:

(1) A DY250 climatic chamber (Angelantoni Test Technologies S.r.l., Massa Martana, Italy), which can provide a temperature range from –40 °C to 180 °C. The temperature in the chamber was measured by means of a Pt100 resistive temperature probe, which was placed at the bottom of the test chamber.
(2) The WinKratos software (Angelantoni Test Technologies S.r.l., Massa Martana, Italy), for remote control of the climatic chamber for automatic tests and temperature profile setting.
(3) A 34461A digital multimeter (Keysight, Santa Rosa, CA, USA) to measure sensor capacitance.
(4) A GDM-8351 digital multimeter (Good Will Instruments Co., Ltd., New Taipei City, Taiwan) to perform 4-wire resistance measurement of a SE019 Pt100 temperature sensor, with an uncertainty of 0.15 °C at 0 °C, which was inserted into the tank to accurately measure liquid temperature.

The temperature profiles were defined by means of the WinKratos software, which allowed remote control of the climatic chamber, and the whole measurement and acquisition process was controlled by the LabVIEW^®^ software (National Instruments Corp., Austin, TX, USA).

**Figure 6:**
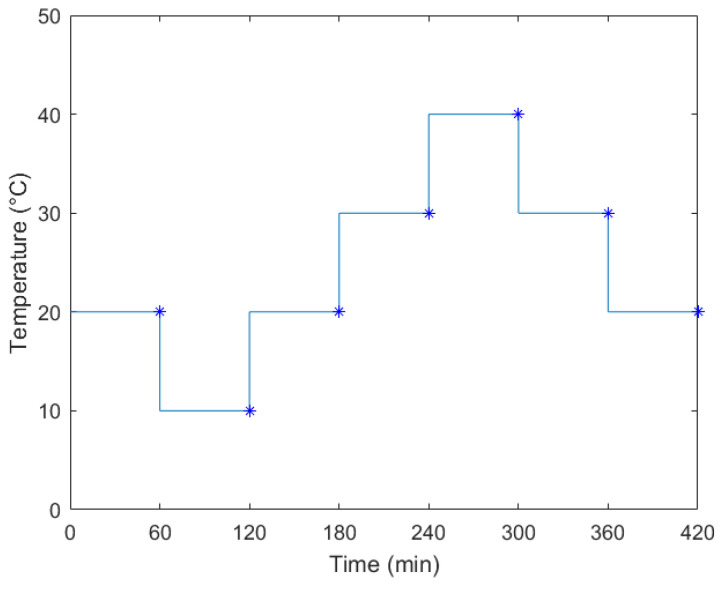
Temperature profile for sensor’s characterization.

**Figure 7:**
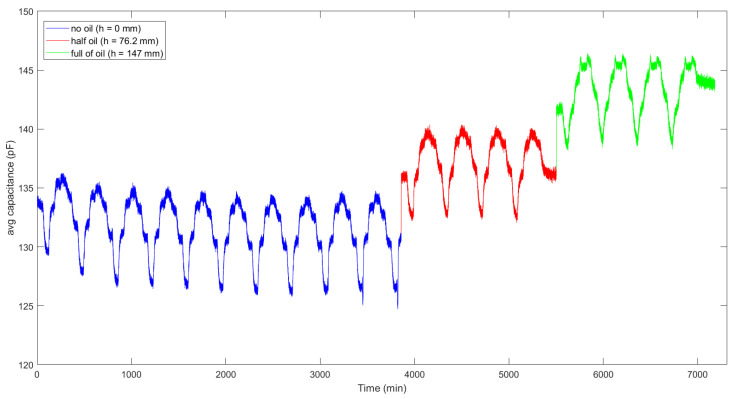
Sensor’s capacitance during consecutive thermal cycles for three oil levels. Initial material settling is observed during the first cycles. A backward moving average with *N* = 100 samples has been performed on capacitance measurements to reduce noise.

The following temperature profile was set: seven steps of 10 °C spanning the range from 10 °C to 40 °C, as shown in Figure 6. Each step lasted 60 min, to ensure liquid temperature stabilization. The slope of the ramp between each step was set as the maximum slope provided by the climatic chamber, and a total of 18 test cycles were performed: the first 10 without filling the tank (*h* = 0 mm), in order to assess stability and to mitigate possible plastic materials settling effects due to alteration of polymer microstructure and relaxation of stresses induced by fabrication, which appeared to have been stabilized after the first six cycles (thus removed from analysis), then four cycles with the tank half filled with oil (*h* = 76.2 mm), and finally four cycles with the tank full of oil (*h* = 147.0 mm). The backward moving average of 100 consecutive capacitance readings was computed for the whole measurement cycle to reduce noise, along with the standard deviation used to quantify the noise. The mean reading time for each capacitance value was 0.29 s. Figure 7 shows the moving average of capacitance readings for the 18 temperature cycles at different levels of liquid. It is evident a positive correlation between capacitance and temperature in the considered temperature range, and between capacitance and liquid level, as already observed in Figure 4. It can also be noted that the first no oil cycles (blue line) differ from the following ones, due to the aforementioned plastic materials settling effects.

**Figure 8:**
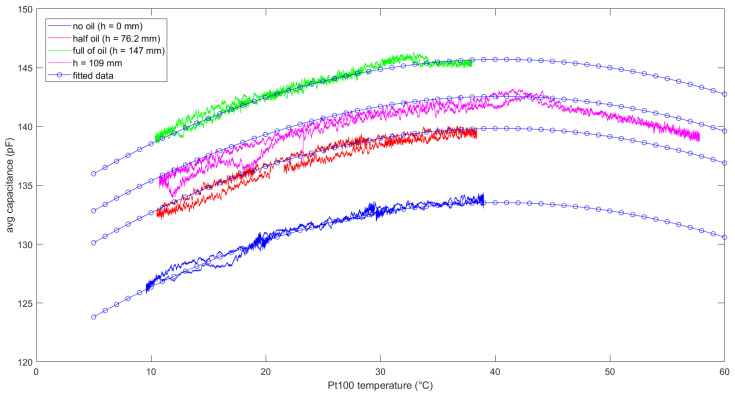
Sensor’s capacitance vs. temperature measured with the Pt100 probe during one thermal cycle for different oil levels. The proposed fitting is also shown. A backward moving average with *N* = 100 samples has been performed on capacitance measurements.

The relationship between capacitance and liquid temperature can be understood with the help of Figure 8, which shows the averaged capacitance of sensor vs. liquid temperature measured by means of the Pt100 sensor (air temperature in case of no oil).

It is suitable to define a function C=f(h,T) which describes the relationship between sensor capacitance *C*, oil level *h*, and air/oil temperature *T*. Different polynomial models have been considered for f(h,T). The following function, with a linear dependence on the liquid level and a quadratic dependence on the temperature, fitted well to the measurement data by assuring both model simplicity and low error model:

(3) $$C=f\left(h\;,\;T\right)=\left(a_{0}T^{2}+a_{1}T+a_{2}\right)+a_{3}h$$

with $a_{0}\;$ = −0.0077 $\mathrm{pF}\cdot {}^{\circ}C^{-2}$, $a_{1}=0.6249\;\mathrm{pF}\cdot {}^{\circ}C^{-1}$, $a_{2}=120.8932\;\mathrm{pF}$, $a_{3}=\;0.0828\;{\mathrm{pF}/\mathrm{mm}}^{-1}$. In fact, each curve presents a maximum at about 40.6 °C, then the correlation between capacitance and temperature becomes negative. Despite the 4-terms model in (3) is quite simple and does not contain cross terms T×h, the goodness of its fitting is comparable to more complex models; for example, the 9-terms polynomial of degree 4 with terms up to $T^{2}h^{2}$ returned a root mean square error (RMSE) of 0.42 pF, $R^{2}\;$= 0.9940, whereas the selected model (3) returned a RMSE of 0.47 pF, with $R^{2}\;$= 0.9926. Finally, to validate Equation (3) for extrapolated data, a further experiment was performed at *h* = 109 mm for a wider temperature range, thus obtaining a 4th experimental curve (in magenta). Two temperature cycles were repeated for this experiment: each cycle consisted of two subsequent ramps, from 10 °C to 60 °C, then down to 10 °C, with a slow slope of 0.33 °C/min. The RMSE between the 4th experimental curve and the extrapolation obtained by applying Equation (3) for *h* = 109 mm is 0.93 pF, with $R^{2}\;$= 0.8298. This is a good result, considering the simplicity of the model. The main parameters considered in sensor characterization, such as sensitivity, repeatability and hysteresis are analysed in the following section.

#### 3.2.1. Sensitivity

The sensor exhibited a nonlinear dependence with temperature. Hence, the sensitivity to temperature changes was calculated as $\frac{d}{dT}f\left(h,T\right)$, for T = 25 °C, which is the middle of the temperature range.

#### 3.2.2. Repeatability

Two main contributions were considered in the evaluation of repeatability: one caused by noise, which is due to the measurement process (directly related to the digital multimeter used to measure capacitance and to thermal noise); the second is due to the difference between consecutive temperature cycles and is related to the intrinsic repeatability of the sensor. Repeatability and noise were evaluated as follows for each level of liquid:

(1) Repeatability between consecutive cycles, due to sensor intrinsic repeatability. It was calculated by considering capacitance measurements ${\bar{c}}_{ijk}$ indexed by: k=1,…,4 corresponding to liquid level $h_{k}$; temperature cycle $j=1,\;\ldots ,\;n_{k}$ (where $n_{k}$ is the number of cycles for a given liquid level); and temperature step i=1,…, 7. Each ${\bar{c}}_{ijk}$ has been calculated as the mean of 100 consecutive capacitance readings taken just before the next temperature change (i.e., when temperature and capacitance is stable) in order to reduce noise (the aforementioned moving average). Therefore, repeatability for a given level of liquid $h_{k}$, has been calculated as follows:
  (4) $$\begin{array}{cc} & i=1,\ldots ,\;7\\ r_{k}=\underset{i}{\mathrm{mean}}\left(\underset{j}{\mathrm{std}}\;{\bar{c}}_{ijk}\right) & j=1,\ldots ,\;n_{k}\\ & k=1,\ldots ,4 \end{array}$$
  where standard deviations are evaluated in sets corresponding to the same temperature step and liquid level, and different temperature cycles.
(2) Noise contribution, due to the measurement process. It was calculated by considering capacitance STD $\sigma_{ijk}$, with the same meaning for indexes as above. Each $\sigma_{ijk}$ was calculated as the STD of 100 consecutive capacitance readings taken just before the next temperature change:

(5) $$\begin{array}{cc} & i=1,\ldots ,\;7\\ \sigma_{k}=\underset{i}{\max}\left(\underset{j}{\mathrm{mean}}\;\sigma_{ijk}\right) & j=1,\ldots ,\;n_{k}\\ & k=1,\ldots ,4 \end{array}$$

When compared with repeatability $r_{k}$, it should be considered that the contribution to the standard deviation of $\;{\bar{c}}_{ijk}$ due to noise is $\;\sigma_{ijk}/\sqrt{N}$, where N=100 is the number of averaged capacitance measurements in $\;{\bar{c}}_{ijk}$.

#### 3.2.3. Hysteresis

The maximum difference between the capacitance measured for increasing and decreasing temperature was calculated to quantify sensor hysteresis: both the increasing and decreasing profiles were fitted with two 2nd-order polynomials, which were used to compute the hysteresis. Finally, the mean value between the $n_{k}$ cycles was considered.

## 4. Discussion

Table 4 summarizes the main parameters obtained from data analysis. It can be noted that the full-scale output (FSO), calculated as the difference between the maximum and minimum of the moving averaged capacitance for each cycle, is almost constant for all the considered liquid levels, suggesting that temperature and liquid level affect capacitance independently.

The sensitivity to oil level variations is 0.078 pF/mm, hence to appreciate a level change of 1 mm, a small capacitance change should be measured. This is not difficult, indeed redout circuits have been proposed which are low-cost, portable, and permit capacitance measurement with 1 fF resolution or impedance measurement in a frequency range up to 50 kHz [25,26]. However, the sensitivity to temperature changes is of 0.24 pF/°C at 25 °C, which means that a variation of 1 °C can cause an error in level measurement of 3 mm. Hence, in sensing liquid level applications, it may be important to measure temperature to compensate undesired variations due to liquid temperature changes, which can be done by using Equation (3). Therefore, it is also necessary to quantify the effect of hysteresis and repeatability on level sensing; to this end, hysteresis and repeatability errors were propagated to temperature and level errors, by using temperature and level sensitivities, as shown in Table 5.

Hysteresis produces a maximum error of 3.5 °C, or equivalently a level error of 10.8 mm. Considering a moving average with *N* = 100, the repeatability error due to measurement noise results in a mean temperature STD of 1.0 °C or, equivalently, a mean level STD of 3.1 mm, which can be furtherly reduced by increasing the averaging points; this is an easy task if we consider slow variations of temperature and liquid level. Moreover, the employment of measurement techniques different from the one used by the digital multimeter (charging and discharging of capacitance through a known resistance) can highly reduce measurement errors. Particular attention should be given to use accurate methods for capacitance measurement in order to reduce this contribution [27]. The repeatability between cycles, quantified by $r_{k}$, is negligible with respect to noise because $r_{k}\ll \sigma_{k}$, as shown in Table 4. Moreover $r_{k}\leq \sigma_{k}/\sqrt{N}$, which means that $r_{k}$ is below detectability and the small observed value can be simply explained as residual noise in $\;{\bar{c}}_{ijk}$, which is the average of N measurements. This is a good result, since this contribution expresses the intrinsic repeatability of the sensor, hence a small $r_{k}$ is a measure of its good performance in level measurement.

Finally, it is important to say that the model obtained by fitting Equation (3) on experimental data presented an RMSE of 0.47 pF, which is mainly due to the noise contribution $\frac{\sigma_{k}}{\sqrt{N}}$ (since the fitting is done on the averaged capacitance) and hysteresis, as shown by curves of Figure 8; therefore, by reducing further the noise contribution as suggested above, the model can be employed to accurately compensate capacitance changes due to temperature by measuring this latter. Purposely designed 3D-printed sensors could be integrated in the same sensor to allow temperature compensation.

## 5. Conclusions

In this paper we have presented a flexible coplanar capacitive sensor for liquid level sensing, 3D-printed by using FFF technology in a single, fully automated, manufacturing process. The sensor has been fabricated by employing commercial materials, allowing fast prototyping with very low cost. It has been characterized with sunflower oil, showing good sensitivity and linearity. Overall, the following conclusions can be drawn:

- Sensitivity, hysteresis and repeatability have been analysed, and their effect on liquid level measurement have been quantified by means of error propagation. The main error contribution is due to random measurement noise, which can be reduced by averaging or by usage of a different capacitance measurement technique.
- The thermal characterization has produced a model relating capacitance with temperature and liquid level, showing a linear dependence on the liquid level and a quadratic dependence on the temperature, with its maximum at about 40.6 °C. The model fitted well to experimental data, providing a valuable tool to compensate errors due to temperature variations, after measuring liquid temperature by means of external or embedded sensors.
- The experiments have shown insensitivity of capacitance to bending stimuli. This may be exploited to develop wearable sensors, not for detecting body motion, but different variables such as temperature or presence of liquids.

We are currently conducting further studies to use this sensor with different liquids. Another possible field which could benefit from the proposed sensor is related to swimming soft robots [28,29], in fact the developed capacitive sensor may be embedded into soft structures to provide feedback related to variables such as liquid temperature or presence/absence of contaminating substances without being affected by robots’ motion.

## Figures and Tables

**Table 1:** Values of sensor’s geometric design parameters.

Parameter	Value
Whole sensor length (mm)	171
Length of sensible part (mm)	148.7
Flexible substrate height (mm)	0.4
Electrodes height (mm)	0.8
Flexible top cover height (mm)	0.4
Electrodes spacing: *s* (mm)	0.8
Electrodes width: *w* (mm)	0.5
Number of electrodes pairs: *N*	114
Length of each electrode: *l* (mm)	25

**Table 2:** Values of process parameters used to print TPU95A and AlfaOhm materials.

Parameter	TPU95A	AlfaOhm
Printing temperature (°C)	223	225
Line width (mm)	0.8	0.4
Printing speed (mm/s)	30	25
Flow (%)	106	110
Fan Speed (%)	50	25

**Table 3:** Capacitance of the sensor when installed on different supports. The mean of 100 capacitance measurements for each bending support is reported. There is no evident change in capacitance when the sensor is not bended with respect to the usage of a bending support.

Support for Sensor Bending	Capacitance (pF)
No bending	124.8
45°	124.8
60°	124.7
120°	124.7
b1	124.8
b2	124.7

**Table 4:** Experimental evaluation, for several oil levels, of: sensitivity to temperature; Full Scale Output (FSO) range during temperature cycles; temperature hysteresis; and repeatability errors due to noise ($\sigma_{k})$, averaged noise ($\sigma_{k}/\sqrt{N}$) and temperature ($r_{k}$).

Oil Level index k	Oil Level $h_{k}\;(\mathrm{mm})$	Sensitivity $\left(\frac{pF}{{}^{\circ}C}\right)\;\;\mathrm{at}\;25\;{}^{\circ}C$	FSO (pF)	Hysteresis		Repeatability					
				(pF)	(% of FSO)	(pF)			(% of FSO)		
						$\sigma_{k}$	$\frac{\sigma_{k}}{\sqrt{N}}$	$r_{k}$	$\sigma_{k}$	$\frac{\sigma_{k}}{\sqrt{N}}$	$r_{k}$
1	0	0.24	10.44	0.43	4.1	2.34	0.23	0.23	22.4	2.2	2.2
2	76.2	“	9.67	0.84	8.7	2.40	0.24	0.18	24.8	2.5	1.9
3	109	“	11.79	0.41	3.5	2.57	0.26	0.15	21.8	2.2	1.3
4	147	“	9.21	0.57	6.2	2.48	0.25	0.25	26.9	2.7	2.7

**Table 5:** Conversion from hysteresis and repeatability capacitance errors to temperature and level errors, evaluated for several oil levels.

Oil Level (mm)	Hysteresis		$\frac{\sigma_{k}}{\sqrt{N}}$	
	Temperature (°C)	Level (mm)	Temperature (°C)	Level (mm)
0	1.8	5.5	1.0	2.9
76.2	3.5	10.8	1.0	3.1
109	1.7	5.3	1.1	3.3
147	2.4	7.3	1.0	3.2

## Data Availability

Not applicable.
